# Comparison of Cranial Ultrasound and Amplitude‐Integrated Electroencephalography in Predicting Neurodevelopmental Outcomes in Neonates With Hypoxic–Ischemic Encephalopathy: A Systematic Review and Meta‐Analysis

**DOI:** 10.1002/jcu.70340

**Published:** 2026-07-17

**Authors:** Meirong Shu, Shilue Zhong, Bo Bao, Dandan Xu, Yingfeng Jiang, Dongxue Lin, Jingzhi Su

**Affiliations:** ^1^ Department of Ultrasound Haikou People's Hospital Haikou China; ^2^ Department of Functional(Electroencephalography Room) Haikou People's Hospital Haikou China

**Keywords:** electroencephalography, hypoxic–ischemic encephalopathy, infant, newborn, ultrasonography

## Abstract

**Background:**

Hypoxic–ischemic encephalopathy (HIE) remains a leading cause of neonatal mortality and long‐term neurodevelopmental impairment.

**Methods:**

PubMed, Embase, Web of Science, and the Cochrane Library were searched from inception to the latest update for observational studies evaluating the prognostic value of aEEG and/or cUS in term and near‐term neonates with HIE. The primary outcome was adverse neurodevelopmental outcome, and the secondary outcome was cerebral palsy (CP). Random‐effects models were applied to estimate pooled proportions and diagnostic performance metrics. Heterogeneity was assessed using the *I* ^2^ statistic, with meta‐regression and sensitivity analyses conducted to explore potential sources of heterogeneity and result robustness.

**Results:**

Ten studies involving 901 neonates with HIE were included. The pooled proportion of adverse neurodevelopmental outcomes (NDO) was 0.44 (95% CI: 0.34–0.54; *I* ^2^ = 84.0%). Subgroup analyses demonstrated similar adverse outcome proportions for aEEG (0.43), cUS (0.42), and conventional EEG‐based patterns (0.50), with no significant between‐group differences. For aEEG, the pooled diagnostic odds ratio for predicting adverse NDO was 18.85 (95% CI: 7.31–48.65). The pooled incidence of CP was 0.02 (95% CI: 0.01–0.07), with low to moderate heterogeneity. Meta‐regression did not identify publication year or assessment timing as significant contributors to heterogeneity, and sensitivity analyses confirmed result stability.

**Conclusions:**

Adverse NDO remain frequent in neonates with HIE. aEEG demonstrates strong prognostic value for early prediction of unfavorable NDO, while cUS provides complementary but less definitive prognostic information. Standardized assessment protocols and outcome definitions are required to improve neuroprognostication in neonatal HIE.

## Graphical abstract

In neonates with hypoxic–ischemic encephalopathy (HIE), adverse neurodevelopmental outcomes (NDO) remain common. Amplitude‐integrated EEG demonstrates strong prognostic value for early prediction, while cranial ultrasound (cUS) provides complementary but less definitive information. Standardized assessment strategies are needed to improve early neuroprognostication in neonatal HIE.

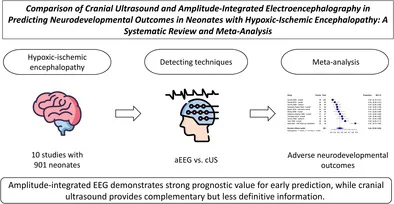

## 1 Introduction

Hypoxic–ischemic encephalopathy (HIE), a type of brain dysfunction that occurs when the brain doesn't receive enough oxygen or blood flow for a period of time, remains one of the leading causes of neonatal mortality and long‐term neurodevelopmental impairment worldwide (Goeral et al. 2017; Steiner et al. 2022). Despite advances in perinatal care and the widespread adoption of therapeutic hypothermia, a substantial proportion of affected neonates continue to experience adverse neurodevelopmental outcomes (NDO), including cognitive impairment, motor dysfunction, and cerebral palsy (CP) (Abiramalatha et al. 2023; Yimenicioglu et al. 2023; Pittet‐Metrailler et al. 2020). Early and reliable prognostic assessment is therefore essential to guide clinical decision‐making, optimize follow‐up strategies, and inform parental counseling during the neonatal period (Cornet et al. 2025; Cavalleri et al. 2014).

Neurophysiological and neuroimaging tools play a central role in the early evaluation of brain injury in neonates with HIE. Amplitude‐integrated electroencephalography (aEEG), a simplified bedside EEG technique, allows continuous monitoring of cerebral background activity and seizure burden during the critical early postnatal period (Glass et al. 2016; Meder et al. 2022). Commonly evaluated aEEG features include background pattern classification (continuous normal voltage [CNV], discontinuous normal voltage [DNV], burst suppression [BS], continuous low voltage [CLV], and flat tracing), the presence and maturity of sleep–wake cycling (SWC), and seizure activity, all of which have been associated with the severity of hypoxic–ischemic brain injury and subsequent NDO. Cranial ultrasound (cUS), in contrast, is widely available, noninvasive, and routinely used to detect structural brain abnormalities, with particular utility in identifying injury to the cortical gray matter (e.g., parasagittal watershed lesions), deep gray matter structures (e.g., basal ganglia and thalamic echogenicity or atrophy), and white matter (e.g., periventricular leukomalacia, increased periventricular echogenicity) (Helbok et al. 2022; Panigrahi et al. 2025). Commonly evaluated cUS injury patterns in HIE include focal or diffuse parenchymal echogenicity, loss of gray–white matter differentiation, thalamic or basal ganglia abnormalities, and ventriculomegaly, all of which have been correlated with neurodevelopmental prognosis.

Previous studies have reported variable associations between abnormal aEEG patterns, ultrasound findings, and subsequent NDO in neonates with HIE (Scoppettuolo et al. 2025; Annink et al. 2020; Boo et al. 2000; Pols et al. 2019). While some investigations suggest that early aEEG abnormalities are strongly predictive of long‐term adverse outcomes, others report limited sensitivity or inconsistent predictive value depending on timing, scoring systems, and outcome definitions (Shankaran 2012). Similarly, the prognostic utility of cUS has been questioned due to its limited sensitivity for detecting early hypoxic–ischemic brain injury compared with advanced neuroimaging techniques such as magnetic resonance imaging (Backman et al. 2017; Mathieson et al. 2024).

Several systematic reviews have addressed neurodevelopmental prognosis in HIE; however, many have focused on a single modality, mixed preterm and term populations, or lacked quantitative synthesis of diagnostic performance metrics. In addition, evolving clinical practices and the increasing use of standardized outcome assessments necessitate an updated synthesis of the available evidence.

Therefore, the present study aimed to systematically review and quantitatively synthesize the evidence on the prognostic value of aEEG and cUS for predicting NDO in term and near‐term neonates with HIE. By integrating pooled incidence estimates, diagnostic performance measures, and exploratory analyses of heterogeneity, this meta‐analysis seeks to provide a comprehensive and clinically relevant assessment of commonly used bedside tools in neonatal neuroprognostication.

## 2 Methods

### 2.1 Study Design and Reporting Standards

This study was conducted as a systematic review and meta‐analysis to evaluate the prognostic value of aEEG and cUS in predicting NDO in neonates with HIE. Specifically, the review question was whether predefined bedside neurophysiological features, including aEEG background pattern, SWC, and seizure activity, and predefined cUS findings, including regional brain injury patterns and injury severity, were associated with adverse NDO in term or near‐term neonates with HIE.

The review was performed in accordance with the preferred reporting items for systematic reviews and meta‐analyses (PRISMA) guidelines (Parums 2021). A predefined protocol was developed prior to literature screening, including eligibility criteria, outcome definitions, and statistical analysis plans. The protocol also specified extraction of index‐test features, operational definitions of abnormal findings, severity classification where available, and harmonized outcome categories to improve comparability across studies.

### 2.2 Data Sources and Search Strategy

A comprehensive and systematic literature search was conducted in four electronic databases: PubMed, Embase, Web of Science, and the Cochrane Library. The databases were searched from inception to February 2026. Only studies published in English were considered. No restrictions were applied with respect to publication year to ensure broad coverage of both early and contemporary evidence.

The search strategy was designed to capture studies evaluating the prognostic value of aEEG and cUS in neonates with HIE. Three core concepts were combined using Boolean operators: (1) HIE or related perinatal hypoxic conditions, (2) neurophysiological or neuroimaging assessment modalities, including amplitude‐integrated EEG, conventional EEG, and cUS, and (3) neurodevelopmental or mortality‐related outcomes. The PubMed search strategy consisted of a combination of Medical Subject Headings and free‐text terms, including “hypoxic ischemic encephalopathy,” “hypoxic ischaemic encephalopathy,” “birth asphyxia,” “amplitude integrated EEG,” “aEEG,” “cUS,” and outcome‐related terms such as “neurodevelopmental outcome,” “prognosis,” “predict*,” “mortality,” and “CP.”

To ensure conceptual consistency across databases, the core search strategy was adapted to the indexing characteristics of each database. In Embase, EMTREE terms were used in combination with corresponding free‐text keywords. In Web of Science, searches were performed using the Topic field to retrieve relevant terms from titles, abstracts, and keywords. The Cochrane Library was searched using free‐text terms aligned with the PubMed strategy. Minor syntax modifications were applied where necessary while preserving identical conceptual frameworks for population, index tests, and outcomes across all databases.

In addition, the reference lists of all included articles and relevant review papers were manually screened to identify potentially eligible studies that may not have been captured through the electronic database searches. The complete search strategies for each database are provided in the Supporting Informations.

### 2.3 Eligibility Criteria

Studies were considered eligible if they met all of the following criteria:

Population: Term or near‐term neonates diagnosed with HIE.

Index tests: Assessment using amplitude‐integrated EEG, cUS, or conventional EEG‐based patterns performed during the neonatal period. For aEEG, eligible studies were required to report at least one prespecified electrophysiological feature, including background pattern, SWC, or seizure activity. For cUS, eligible studies were required to report at least one predefined regional or pattern‐based structural abnormality, including cortical gray matter injury, deep gray matter injury, white matter injury, parenchymal echogenicity, loss of gray–white matter differentiation, periventricular leukomalacia, ventriculomegaly, cystic change, or atrophy. Studies evaluating conventional EEG or automated EEG‐derived background features were included when these findings were used as neonatal neurophysiological prognostic markers.

Outcomes: Reported NDO, including adverse NDO or CP, assessed through clinical diagnosis or standardized follow‐up evaluations. Eligible studies had to provide extractable data allowing classification of outcomes as favorable versus adverse, or provide sufficient information to derive raw counts of adverse NDO, CP, or death.

Study design: Original observational studies, including prospective or retrospective cohort studies. Secondary analyses of randomized trials or multicenter cohorts were also eligible if the index‐test findings and NDO were extractable.

Studies were excluded if they met any of the following conditions:

Review articles, editorials, conference abstracts, or other non‐original research designs.

Studies without extractable neurodevelopmental outcome data.

Studies in which the index test was not performed during the neonatal period, studies without sufficient information on the definition of abnormal aEEG, cUS, EEG, or outcome status, and studies in which outcomes could not be dichotomized as favorable versus adverse were excluded from quantitative synthesis.

### 2.4 Study Selection

All retrieved records were imported into reference management software, and duplicate records were removed.

Two reviewers independently screened titles and abstracts for potential eligibility. Full‐text articles were subsequently retrieved and assessed for inclusion based on the predefined criteria. Any discrepancies during the selection process were resolved through discussion or consultation with a third reviewer.

### 2.5 Data Extraction

Data extraction was performed independently by two reviewers using a standardized data extraction form. Extracted information included:

Study characteristics (author, publication year, study design, and setting).

Population characteristics (sample size and diagnostic criteria for HIE).

Details of index tests (type of modality and timing of assessment).

Definitions and assessment methods of NDO.

Specific aEEG features assessed, including background pattern, SWC, and seizure activity.

Specific cUS brain regions evaluated, including cortical gray matter, deep gray matter, and white matter.

Specific cUS injury patterns assessed, including parenchymal echogenicity, loss of gray–white matter differentiation, thalamic or basal ganglia abnormalities, periventricular leukomalacia, ventriculomegaly, cystic evolution, and atrophy.

Original study definitions of abnormal index‐test findings, severity grades, diagnostic thresholds, and outcome categories.

Raw numbers of neonates with favorable outcomes, adverse NDO, CP, or death whenever available.

When multiple time points or diagnostic thresholds were reported, data corresponding to clinically relevant early neonatal assessments were preferentially extracted. For aEEG, early recordings within the first 6–72 h after birth were prioritized, depending on the timing reported in each study. For cUS, scans performed during the first postnatal week were prioritized because this period corresponds to early structural assessment of hypoxic–ischemic injury. When several thresholds or repeated measurements were reported, the threshold prespecified by the original study as prognostically relevant was preferentially extracted. If more than one eligible time point was available, we selected the time point most closely aligned with the intended early prognostic use of the index test. Disagreements were resolved by consensus.

To improve transparency, the specific index‐test features, original definitions of abnormality or severity, and harmonized categories used for synthesis are summarized in Table S1. Study‐specific outcome definitions, follow‐up timing, and harmonized adverse outcome categories are summarized in Table S2.

### 2.6 aEEG Feature Definitions

Given the heterogeneity in aEEG reporting across included studies, we predefined the aEEG features of interest based on established classification systems. These included: (i) background pattern, categorized according to the Hellström‐Westas criteria as CNV, DNV, BS, CLV, or flat (isoelectric) tracing; (ii) SWC, classified as present/absent or immature/mature; and (iii) seizure activity, identified as recurrent epileptiform discharges on the raw EEG with corresponding changes in the aEEG trace. Studies reporting any of these features were eligible for inclusion. When multiple features were reported, background pattern was prioritized for diagnostic performance synthesis, as it represents the most consistently validated prognostic marker.

For data harmonization, abnormal aEEG was operationally defined as severely abnormal background activity, including BS, CLV, or flat/isoelectric tracing, or as an abnormal aEEG classification according to the threshold prespecified in each original study. CNV and DNV with recovery over time were generally considered favorable or non‐severe patterns. SWC and seizure activity were extracted when reported; however, because these features were inconsistently reported across studies, they were used primarily for descriptive synthesis and interpretation unless feature‐specific diagnostic data were available. When several aEEG features were reported in the same cohort, background pattern was prioritized for quantitative diagnostic synthesis to avoid duplicate counting of participants.

For studies reporting conventional EEG or automated EEG‐derived background features rather than aEEG, abnormal background activity, BS network features, or quantitative EEG abnormalities were extracted according to the original study definitions and analyzed as a separate EEG‐based subgroup rather than being combined directly with aEEG diagnostic performance estimates.

### 2.7 Cranial Ultrasound Evaluation Criteria

Given the variability in cUS reporting across studies, we predefined the brain regions and injury patterns of interest. Evaluated brain regions included: (i) cortical gray matter (parasagittal watershed zones, cortical mantle); (ii) deep gray matter (thalami, basal ganglia, hippocampi); and (iii) white matter (periventricular white matter, corpus callosum). Evaluated injury patterns included: (a) parenchymal echogenicity (focal or diffuse increased echogenicity); (b) loss of gray–white matter differentiation; (c) thalamic and/or basal ganglia abnormalities (increased echogenicity, atrophy, or cystic changes); (d) periventricular leukomalacia (increased periventricular echogenicity with or without cystic evolution); and (e) ventriculomegaly or other structural abnormalities. Studies reporting any of these regional or pattern‐based assessments were eligible for inclusion.

### 2.8 Cranial Ultrasound Severity Classification and Data Harmonization

Because cUS severity grading differed across the included studies, we harmonized reported findings into a pragmatic severity framework based on the extent, anatomical location, persistence, and destructive nature of injury. A normal cUS was defined as the absence of abnormal parenchymal echogenicity, preserved gray–white matter differentiation, no deep gray matter abnormality, no cystic change, and no ventriculomegaly. Mild abnormalities included focal or transient increased echogenicity without deep gray matter involvement, cystic evolution, atrophy, or ventricular enlargement. Moderate abnormalities included persistent or diffuse parenchymal echogenicity, impaired gray–white matter differentiation, periventricular white matter injury, or isolated thalamic/basal ganglia echogenicity without extensive tissue loss. Severe abnormalities included extensive bilateral parenchymal echogenicity, marked loss of gray–white matter differentiation, deep gray matter injury involving the thalami or basal ganglia, cystic evolution, cerebral atrophy, or ventriculomegaly suggesting substantial tissue injury.

For quantitative synthesis and interpretation, cUS findings were dichotomized as non‐severe/favorable imaging findings versus adverse/severe imaging findings when sufficient detail was available. Normal or mild findings were classified as non‐severe, whereas moderate or severe abnormalities were classified as adverse/severe. When an original study reported only “normal” versus “abnormal” cUS without further severity grading, the study‐specific definition was retained and recorded as ungraded abnormal cUS. When a structured cUS score was reported, the original study threshold for clinically relevant or prognostically adverse injury was used, and the corresponding category was mapped to the harmonized adverse/severe imaging group. These harmonized categories were used for data extraction and interpretation, while variability in original cUS scoring systems was considered a source of methodological heterogeneity.

Because cUS was less frequently reported than aEEG across the included studies, detailed cUS regions, injury patterns, original severity definitions, and harmonized categories were tabulated separately in Table S1.

### 2.9 Quality Assessment

The methodological quality of the included studies was assessed independently by two reviewers using the Quality Assessment of Diagnostic Accuracy Studies 2 (QUADAS‐2) tools (Savović et al. 2018). For each domain, the risk of bias was judged as low, high, or unclear based on predefined signaling questions. Specifically, patient selection was evaluated with respect to the use of consecutive or representative sampling and the avoidance of inappropriate exclusions. The index test domain assessed whether amplitude‐integrated EEG or cUS results were interpreted without knowledge of outcome status and whether prespecified diagnostic criteria were applied. For the index test domain, we also considered whether the original study clearly described the specific aEEG, EEG, or cUS feature assessed, whether abnormality or severity thresholds were prespecified, and whether interpretation of the index test was independent of outcome status. The reference standard domain examined the appropriateness and independence of neurodevelopmental outcome assessment, including the use of standardized clinical diagnoses or validated follow‐up evaluations. For the reference standard domain, we considered whether adverse neurodevelopmental outcome was defined using standardized developmental instruments, clinical neurological examination, CP diagnosis, death, or a clearly stated composite endpoint. The flow and timing domain evaluated whether all enrolled participants were included in the analysis and whether the interval between index testing and outcome assessment was clinically appropriate.

Key domains evaluated included participant selection, index test assessment, outcome ascertainment, and completeness of follow‐up. Each domain was judged as having low, unclear, or high risk of bias. Discrepancies in quality assessment were resolved through discussion. Studies with unclear or heterogeneous definitions of index‐test abnormality or outcome status were not excluded solely on this basis if extractable data were available, but this was reflected in the risk‐of‐bias assessment and considered when interpreting heterogeneity.

### 2.10 Outcome Definitions

The primary outcome was adverse neurodevelopmental outcome, defined as unfavorable neurological or developmental status assessed during follow‐up. This composite outcome encompassed cognitive impairment (e.g., developmental quotient or intelligence quotient > 1 or 2 standard deviations below the normative mean), motor dysfunction (e.g., abnormal motor scores on standardized assessments), global developmental delay (e.g., failure to meet age‐appropriate milestones), severe neurosensory impairment (blindness or deafness), or death attributable to HIE, as reported in the original studies. Given heterogeneity in assessment tools across studies—including the Bayley Scales of Infant and Toddler Development (BSID‐II/III/IV), Griffiths Mental Development Scales, and clinical neurological examination—we operationally classified outcomes as “favorable” versus “adverse” based on each study's predefined thresholds. Where studies reported multiple follow‐up time points, the latest available assessment was preferentially extracted to maximize prognostic validity.

The secondary outcome was CP, defined according to clinical diagnostic criteria reported in the original studies, typically encompassing non‐progressive disorders of movement and posture attributed to early brain injury. Studies using the Surveillance of Cerebral Palsy in Europe (SCPE) definition or comparable standardized clinical criteria were included.

To enable cross‐study comparability, we extracted raw counts of adverse versus favorable outcomes rather than relying on study‐specific composite scores. When studies reported outcomes using graded severity scales (e.g., mild/moderate/severe disability), we dichotomized these into adverse (moderate/severe disability or death) versus favorable (normal/mild disability) categories, consistent with prior meta‐analyses in neonatal encephalopathy.

For studies using Bayley Scales, adverse outcome was defined according to the cutoff prespecified by the original study, most commonly a Mental Development Index, Psychomotor Development Index, cognitive composite score, motor composite score, or language composite score below the study‐defined threshold. Where studies used clinical neurological examination, adverse outcome was defined as moderate or severe neurological impairment, CP, global developmental delay, severe neurosensory impairment, or death. Mild developmental delay or mild neurological abnormality without functional impairment was classified as favorable when the original study grouped it with normal outcome. Death was included in the adverse outcome category when the original study considered death part of the composite endpoint or when death was attributable to HIE.

Therefore, the pooled estimate should be interpreted as a harmonized composite measure of unfavorable neurodevelopmental status after HIE, rather than as an estimate based on a single uniform developmental instrument, identical follow‐up age, or identical disability threshold across all studies. Study‐specific outcome definitions and their harmonized categories are shown in Table S2.

### 2.11 Statistical Analysis

Meta‐analyses were performed using random‐effects models to account for anticipated clinical and methodological heterogeneity among studies. Pooled proportions were calculated for adverse NDO and CP. Given that included studies employed diverse assessment tools, follow‐up durations, and outcome thresholds, we pooled study‐level proportions (number of adverse outcomes divided by total assessed neonates) rather than individual patient data. This approach allows integration of studies with varying outcome definitions while acknowledging that the underlying construct of “adverse neurodevelopmental outcome” may differ across settings. Accordingly, pooled adverse outcome proportions were interpreted as estimates of the overall burden of harmonized adverse neurodevelopmental outcome after HIE, not as estimates of a single identical clinical endpoint.

For diagnostic performance of aEEG, pooled diagnostic odds ratios and sensitivity estimates were synthesized when sufficient data were available. Diagnostic performance analyses were restricted to studies providing extractable two‐by‐two data or sufficient information to derive true‐positive, false‐positive, true‐negative, and false‐negative counts. When multiple aEEG features were available from the same cohort, background pattern was used as the primary index‐test variable for diagnostic synthesis, because it was the most consistently reported and clinically validated aEEG feature. SWC and seizure activity were summarized descriptively unless feature‐specific diagnostic data were available. To compare prognostic performance across modalities (aEEG versus cUS versus conventional EEG), we analyzed the pooled proportion of adverse outcomes within each subgroup and tested for between‐group differences using meta‐regression with a permutation test, recognizing that direct head‐to‐head comparisons within the same patient cohort were rarely available. These subgroup comparisons were therefore interpreted as indirect exploratory comparisons rather than direct within‐patient comparisons of test accuracy.

Between‐study heterogeneity was assessed using the Cochran Q test and quantified with the *I* ^2^ statistic, where *I* ^2^ < 25% indicated low heterogeneity, 25%–75% moderate heterogeneity, and > 75% high heterogeneity. Clinical and methodological sources of heterogeneity considered a priori included differences in aEEG features assessed, cUS brain regions and severity thresholds, timing of neonatal assessment, use of therapeutic hypothermia, follow‐up duration, developmental assessment tools, and study‐specific definitions of adverse outcome.

Predefined subgroup analyses were conducted according to the type of index test modality. Additional subgroup analyses stratified by outcome assessment timing (≤ 12 months versus > 12 months corrected age) and outcome assessment method (standardized developmental scales versus clinical examination alone) were planned to explore whether these factors contributed to observed heterogeneity. Where data permitted, additional exploratory analyses were planned according to whether adverse outcome was defined using standardized developmental testing versus composite clinical endpoints, and whether imaging or neurophysiological abnormality was graded using prespecified severity categories versus binary normal/abnormal classification.

Meta‐regression analyses were planned to explore potential sources of heterogeneity, including publication year, assessment time point, and outcome assessment methodology. Because of the limited number of included studies, meta‐regression results were considered exploratory and hypothesis‐generating. We avoided overinterpretation of nonsignificant meta‐regression findings when the number of studies in a subgroup was small.

Publication bias was evaluated through visual inspection of funnel plots when at least 10 studies were included in a meta‐analysis. Formal small‐study effect tests were not emphasized when fewer than 10 studies contributed to an analysis because such tests have limited power and may be misleading in small meta‐analyses.

Sensitivity analyses were performed using a leave‐one‐out approach to assess the robustness of pooled estimates. Additional sensitivity analyses restricted to studies using standardized developmental assessment tools (e.g., Bayley Scales) were planned to evaluate whether outcome measurement heterogeneity influenced pooled estimates. When feasible, sensitivity analyses were also planned by excluding studies that used neonatal MRI injury rather than long‐term neurodevelopmental assessment as part of the reference standard, and by excluding studies with ungraded or poorly defined index‐test abnormalities. These analyses were interpreted cautiously because the number of eligible studies was limited.

## 3 Results

### 3.1 Study Selection and Study Characteristics

The study selection process is summarized in Figure 1. A total of 2200 records were identified through database searches, including PubMed (*n* = 640), Embase (*n* = 918), Web of Science (*n* = 581), and the Cochrane Library (*n* = 61). After removal of 1080 duplicate records, 1120 records remained for title and abstract screening, of which 1040 were excluded.

**FIGURE 1:**
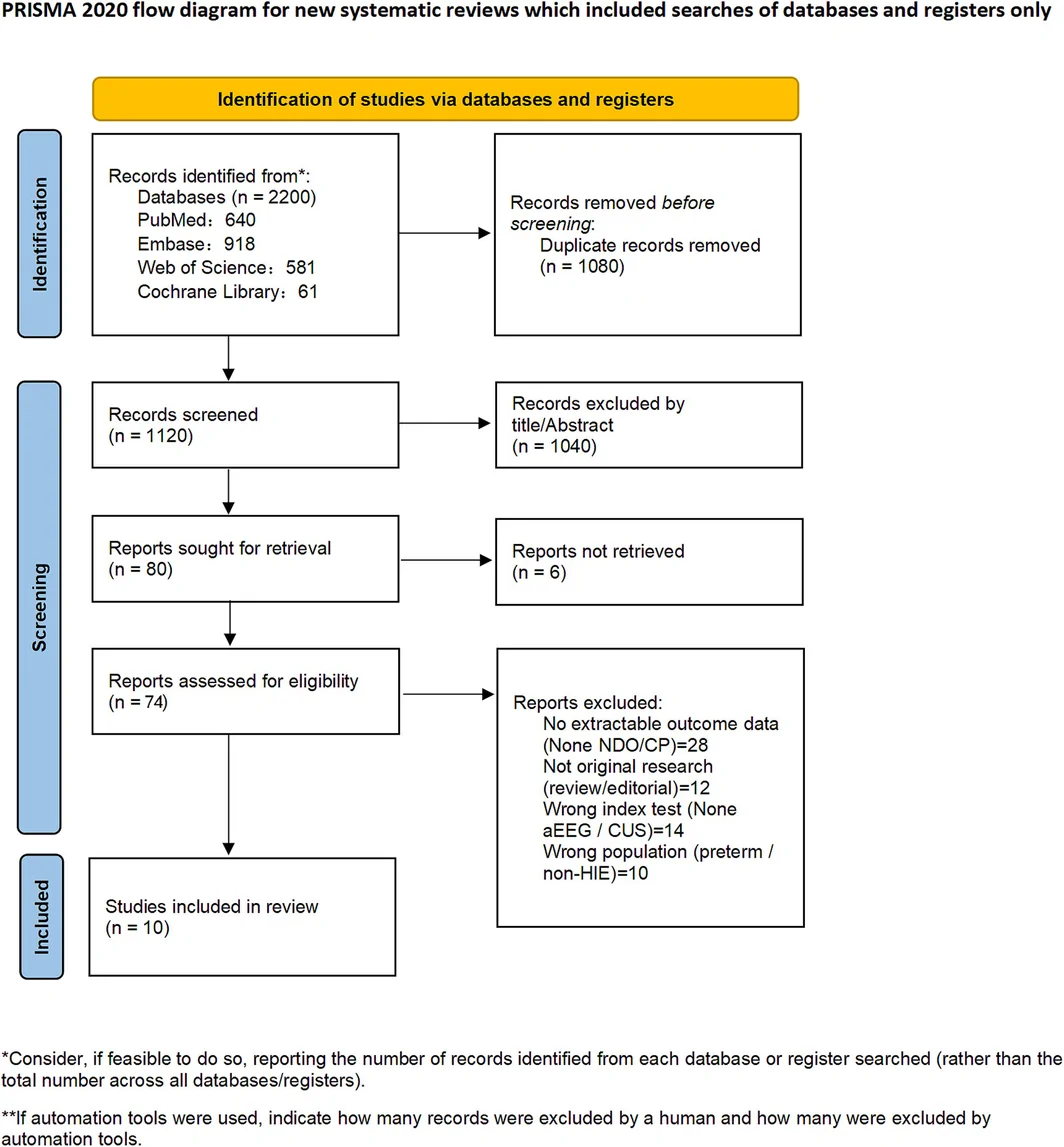
PRISMA flow diagram of study selection.

Eighty full‐text articles were sought for retrieval, and six reports could not be retrieved. Consequently, 74 full‐text articles were assessed for eligibility. Among these, 64 studies were excluded due to lack of extractable neurodevelopmental outcome data (*n* = 28), non‐original study design such as reviews or editorials (*n* = 12), inappropriate index tests without amplitude‐integrated EEG (aEEG) or cUS (*n* = 14), or ineligible populations including preterm infants or non–HIE cases (*n* = 10). Ultimately, 10 studies met the inclusion criteria and were included in the systematic review and meta‐analysis.

The main characteristics of the included studies are summarized in Table 1 (Cornet et al. 2025; Meder et al. 2022; Annink et al. 2020; Hellström‐Westas et al. 1995; Toet et al. 1999; Shany et al. 2006; Thoresen et al. 2010; Sewell et al. 2018; Gonzalez‐Tamez et al. 2025; Kota et al. 2024). The studies were published between the mid‐1990s and 2025 and predominantly employed prospective or retrospective cohort designs. All included studies enrolled term or near‐term neonates diagnosed with HIE. The index tests evaluated included amplitude‐integrated EEG, cUS, or conventional EEG‐based patterns, with assessment time points ranging from the first hours after birth to several days of life. For aEEG studies, the most commonly extracted feature was background pattern, including CNV, DNV, BS, CLV, or flat/isoelectric tracing. SWC and seizure activity were extracted when reported, but these features were less consistently available for quantitative synthesis. For the cUS subgroup, extracted findings included predefined regional and pattern‐based abnormalities involving cortical gray matter, deep gray matter, and white matter, including parenchymal echogenicity, loss of gray–white matter differentiation, thalamic or basal ganglia abnormalities, periventricular leukomalacia, ventriculomegaly, cystic evolution, and atrophy.

**TABLE 1:** Study characteristics.

Study (Year)	Country/Region	Study design	Sample size (n)	HIE severity	Index test	Timing of test	Reference standard	Follow‐up
Hellström‐Westas et al. 1995	Sweden	Prospective cohort	47	Moderate–severe	aEEG	≤ 6 h after birth	Death or neurological sequelae (incl. CP)	12–72 months
Toet et al. 1999	Netherlands/Sweden	Prospective cohort	68	Mild–severe	aEEG	3 and 6 h	Death, CP, or global delay (DQ < 80)	12 months–6 years
Shany et al. 2006	Israel	Prospective cohort	39	Mild–severe	aEEG	≤ 6 h	Death or poor combined neurodevelopmental outcome	≥ 30 months
Thoresen et al. 2010	Multicenter (Europe)	Randomized trial (secondary analysis)	74	Moderate–severe	aEEG	3–6 h	Death or severe disability (Bayley‐II, GMFCS)	18 months
Annink et al. 2020	Netherlands	Prospective cohort	83/35	Moderate–severe	Cranial ultrasound (cUS)	First postnatal week	Death, CP, or BSITD‐III < 85	24 months
Sewell et al. 2018	USA	Prospective cohort	80	Moderate–severe	aEEG	≤ 72 h (evolution)	Death or severe MRI injury	Neonatal–MRI
Meder et al. 2022	USA	Prospective cohort	149	Moderate–severe	aEEG	First 24 h	Death or Bayley‐II MDI/PDI < 70	24 months
Cornet et al. 2025	USA (HEAL)	Multicenter cohort (secondary analysis)	203	Moderate–severe	EEG/BSN	≥ 2 h after EEG start	Death or severe NDI (Bayley‐III, GMFCS)	22–36 months
Gonzalez‐Tamez et al. 2025	Sweden	Population‐based cohort	85	Moderate–severe	EEG/BSN	6–12 h (early)	Moderate–severe NDI or death	24 months
Kota et al. 2024	USA	Prospective cohort	38	Mild–severe	EEG/BSN	6–9 h	Death or Bayley‐III < 85	24 months

NDO were assessed using standardized clinical diagnoses or follow‐up evaluations, including adverse NDO and CP. Study‐specific index‐test definitions, imaging features, severity classifications, and harmonized categories are summarized in Table S1. Study‐specific outcome definitions, follow‐up timing, and harmonized adverse outcome categories are summarized in Table S2.

### 3.2 Methodological Quality of Included Studies

The methodological quality assessment of the included studies is presented in Table 2. Overall, the studies demonstrated moderate methodological quality. Most studies clearly defined the study population and index tests and reported prespecified NDO. Variability was observed in outcome assessment methods and follow‐up duration. Additional variability was also noted in the level of detail used to define abnormal index‐test findings. In particular, aEEG studies differed in whether they reported background pattern alone or additional features such as SWC and seizure activity, while cUS reporting differed with respect to anatomical regions assessed, injury patterns described, and severity thresholds applied. Across studies, the risk of bias related to participant selection and outcome ascertainment was generally acceptable, supporting the inclusion of these studies in the quantitative synthesis. However, studies with incomplete or heterogeneous definitions of index‐test abnormality or outcome status were considered to have moderate risk in the relevant QUADAS‐2 domains, and this variability was considered when interpreting heterogeneity.

**TABLE 2:** Methodological quality of included studies.

Study	Patient selection	Index test	Reference standard	Flow and timing	Overall risk
Hellstrom‐Westas 1995	Low	Low	Low	Low	Low
Toet 1999	Low	Low	Low	Low	Low
Shany 2006	Low	Low	Low	Moderate	Moderate
Thoresen 2010	Low	Low	Low	Low	Low
Annink 2020	Low	Low	Low	Low	Low
Sewell 2018	Low	Moderate	Moderate	Low	Moderate
Meder 2022	Low	Moderate	Moderate	Low	Moderate
Cornet 2025	Low	Moderate	Low	Low	Moderate
Gonzalez‐Tamez 2025	Low	Moderate	Moderate	Low	Moderate
Kota 2024	Low	Moderate	Moderate	Moderate	Moderate

### 3.3 Summary of Index‐Test Findings and Harmonized Categories

Across the included aEEG studies, abnormal background pattern was the most consistently reported prognostic feature and therefore served as the primary aEEG variable for diagnostic performance synthesis. Severely abnormal background patterns, including BS, CLV, and flat/isoelectric tracing, were harmonized as adverse or prognostically abnormal aEEG findings. CNV and DNV with recovery over time were generally classified as favorable or non‐severe patterns. SWC and seizure activity were extracted when available but were not uniformly reported across studies; therefore, these features were mainly used to support descriptive interpretation rather than pooled feature‐specific diagnostic analyses.

For cUS, the available studies reported structural injury findings using study‐specific scoring systems or descriptive ultrasound abnormalities. These findings were harmonized according to the predefined framework described in the Methods. Normal or mild abnormalities, such as focal or transient echogenicity without deep gray matter involvement or destructive change, were classified as non‐severe. Moderate or severe abnormalities, including persistent or diffuse parenchymal echogenicity, impaired gray–white matter differentiation, deep gray matter injury involving the thalami or basal ganglia, cystic evolution, cerebral atrophy, or ventriculomegaly, were classified as adverse or severe imaging findings. Because detailed cUS data were less frequently available than aEEG data, cUS findings were interpreted as complementary structural prognostic information rather than as a directly equivalent comparator to aEEG.

### 3.4 Incidence of Adverse Neurodevelopmental Outcomes

The pooled proportion of adverse NDO is shown in Figure 2. A total of 901 neonates were included across the contributing studies. The random‐effects meta‐analysis yielded a pooled adverse outcome proportion of 0.44 (95% CI: 0.34–0.54). Substantial heterogeneity was observed (*I* ^2^ = 84.0%, *p* < 0.0001), reflecting variability in outcome definitions, assessment tools, and follow‐up durations across studies. Because adverse neurodevelopmental outcome was defined differently across the included studies, this pooled estimate should be interpreted as a harmonized composite measure of unfavorable neurological or developmental status after HIE rather than as an estimate based on a single uniform developmental instrument, follow‐up age, or disability threshold. Differences in cUS severity thresholds, aEEG reporting, and outcome definitions likely contributed to the observed heterogeneity.

**FIGURE 2:**
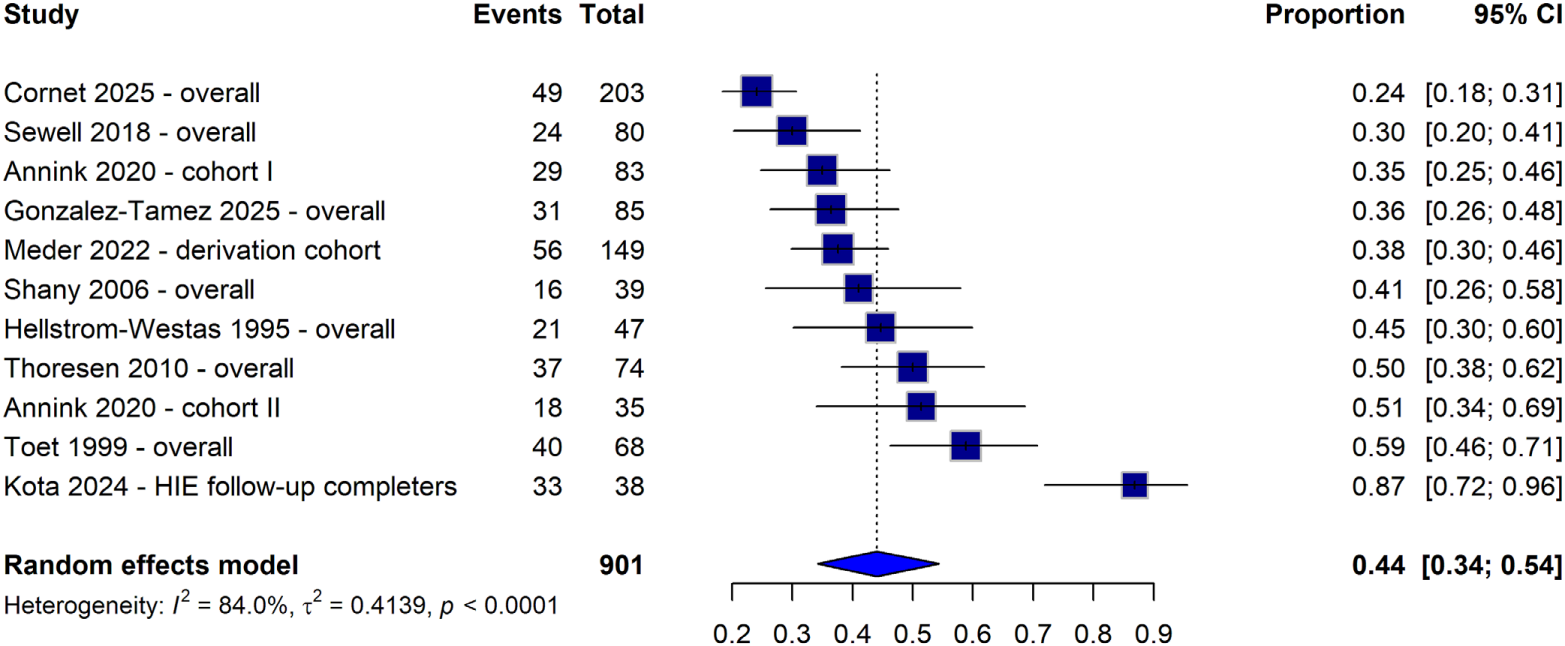
Forest plot of pooled proportion of adverse neurodevelopmental outcomes in neonates with hypoxic–ischemic encephalopathy.

Subgroup analysis stratified by index test modality is shown in Figure S1. The pooled proportion of adverse outcomes was 0.43 (95% CI: 0.35–0.52) for amplitude‐integrated EEG, 0.42 (95% CI: 0.27–0.58) for cUS, and 0.50 (95% CI: 0.15–0.86) for conventional EEG or BS patterns. No statistically significant differences were observed between subgroups (test for subgroup differences, *p* = 0.9379), although this finding should be interpreted cautiously because the subgroup comparison was indirect and based on study‐level estimates rather than direct head‐to‐head comparisons within the same patient cohorts. Therefore, the absence of a statistically significant subgroup difference does not establish equivalence between modalities but suggests that adverse outcomes were frequent across studies evaluating neurophysiological or ultrasound‐based prognostic markers.

### 3.5 Diagnostic Performance of Amplitude‐Integrated EEG

The diagnostic performance of amplitude‐integrated EEG for predicting adverse NDO in neonates with HIE is shown in Figure 3. Individual studies reported diagnostic odds ratios across different assessment time points. Using a random‐effects model, the pooled diagnostic odds ratio was 18.85 (95% CI: 7.31–48.65), indicating a strong overall association between abnormal aEEG findings and adverse NDO. Moderate heterogeneity was observed among studies (*I* ^2^ = 61.3%, *p* = 0.0168). This analysis primarily reflected abnormal aEEG background pattern, because background classification was the most consistently reported aEEG feature across studies. SWC and seizure activity were not pooled separately because feature‐specific diagnostic data were inconsistently available.

**FIGURE 3:**
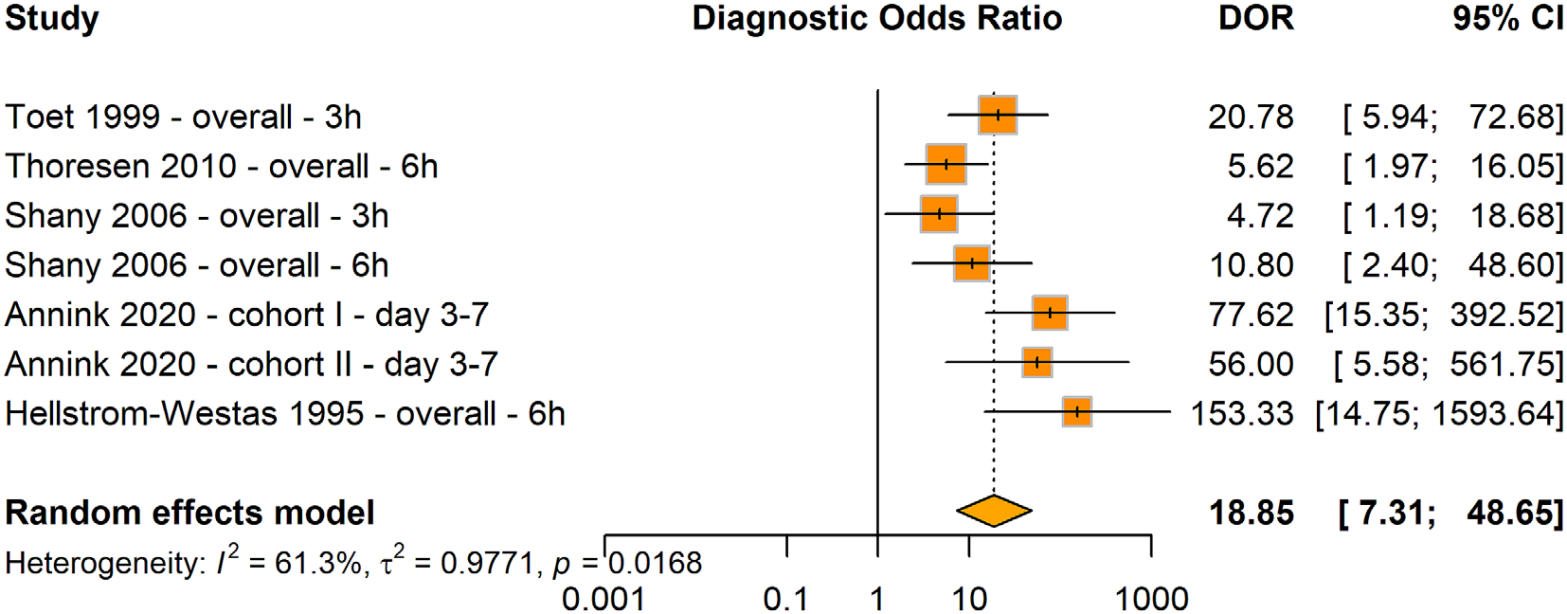
Diagnostic performance of amplitude‐integrated electroencephalography for predicting adverse neurodevelopmental outcomes.

### 3.6 Incidence of Cerebral Palsy

The pooled incidence of CP among neonates with HIE is presented in Figure 4. Four studies contributed data to this analysis. The pooled proportion of CP, calculated using a random‐effects model, was 0.02 (95% CI: 0.01–0.07). Heterogeneity across studies was low to moderate (*I* ^2^ = 39.7%, *p* = 0.1737). Compared with the broader composite adverse neurodevelopmental outcome, CP represented a more specific clinical endpoint and was reported in fewer studies, which limited the precision of this estimate and reduced the ability to explore sources of heterogeneity.

**FIGURE 4:**
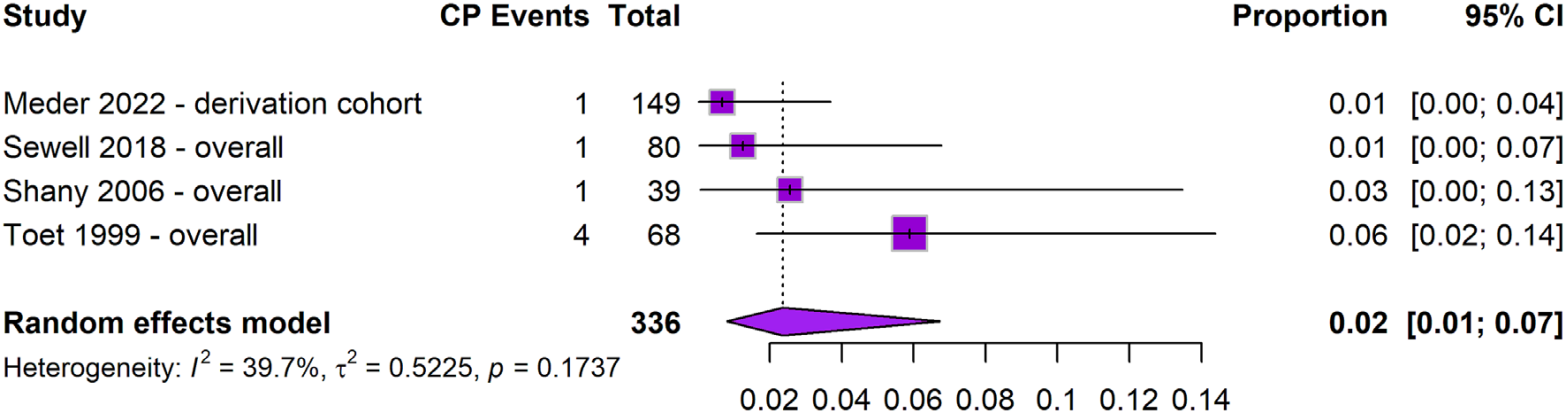
Forest plot of pooled incidence of cerebral palsy in neonates with hypoxic–ischemic encephalopathy.

### 3.7 Meta‐Regression Analyses

Meta‐regression analyses exploring potential sources of heterogeneity are presented in Figure 5. Publication year was not significantly associated with the proportion of adverse NDO (*β* = −0.0113, *p* = 0.602). Similarly, no significant association was identified between assessment time point and diagnostic sensitivity (*β* = 0.0038, *p* = 0.498). For CP, a borderline inverse association with publication year was observed (*β* = −0.0623, *p* = 0.082), although this did not reach statistical significance.

**FIGURE 5:**
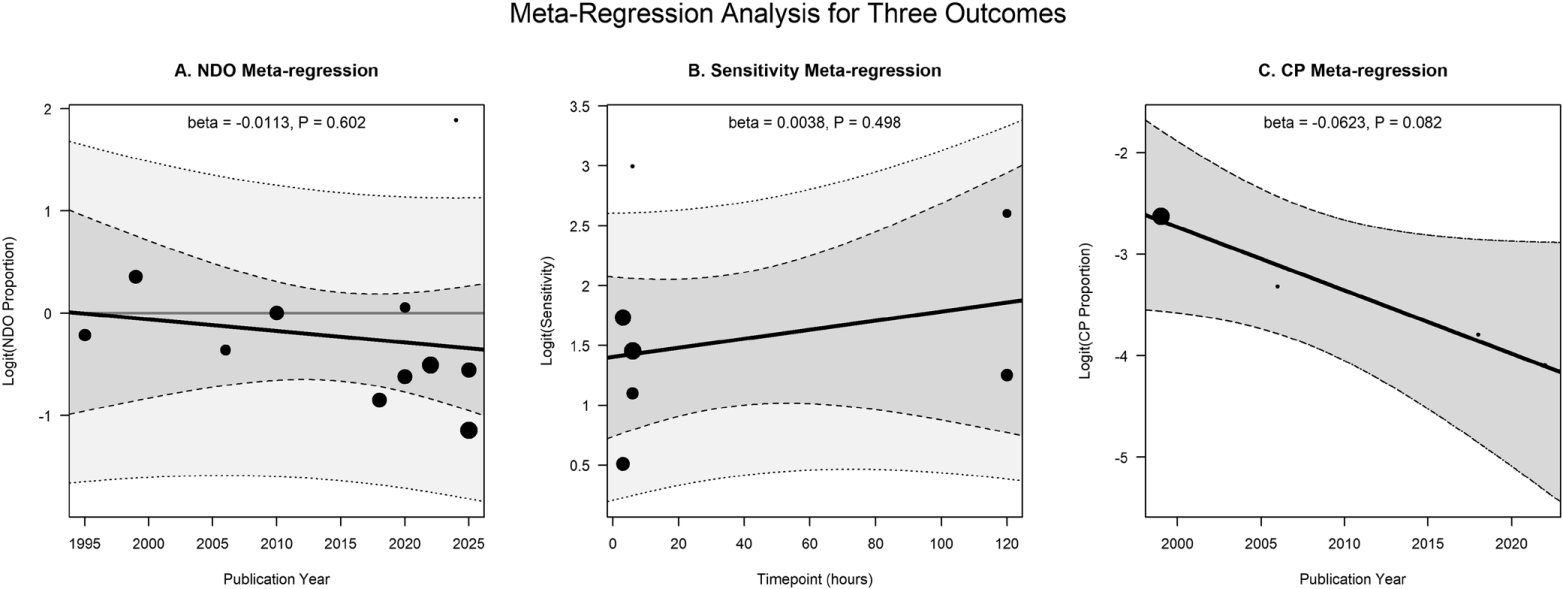
Meta‐regression analyses exploring sources of heterogeneity. (A) Meta‐regression analysis of publication year and adverse neurodevelopmental outcomes. (B) Meta‐regression analysis of assessment timepoint and sensitivity. (C) Meta‐regression analysis of publication year and cerebral palsy incidence. The solid line represents the fitted meta‐regression line, and dashed lines indicate the confidence bands.

### 3.8 Publication Bias

Visual inspection of the funnel plots (Figure 6) revealed that the plot for adverse NDO was approximately symmetrical, although a few studies were unevenly distributed at the lower precision region. In contrast, the funnel plots for diagnostic sensitivity and CP showed some degree of asymmetry, particularly in the small‐study region, where studies tended to cluster on one side of the pooled effect. These patterns suggest the possible presence of small‐study effects, although the limited number of included studies reduces the reliability of visual interpretation.

**FIGURE 6:**
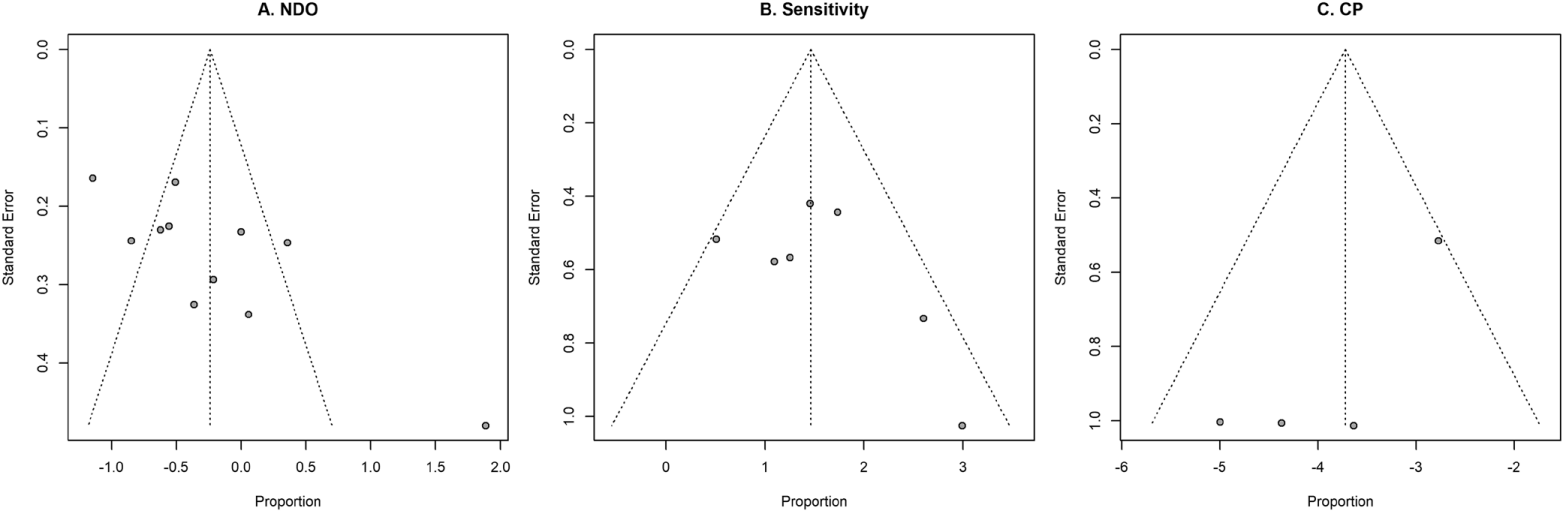
Funnel plots assessing publication bias for primary and secondary outcomes. (A) Funnel plot for adverse neurodevelopmental outcomes. (B) Funnel plot for sensitivity of amplitude‐integrated electroencephalography. (C) Funnel plot for cerebral palsy incidence. The vertical dashed line represents the pooled effect estimate, and the diagonal dashed lines represent the expected 95% confidence limits.

### 3.9 Sensitivity Analyses

Leave‐one‐out sensitivity analyses were conducted to evaluate the robustness of the pooled estimates.

The pooled proportion of adverse NDO also remained consistent following sequential study exclusion, with estimates ranging from 40% to 46% (Figure S2).

For diagnostic sensitivity of amplitude‐integrated EEG (Figure S3), sequential exclusion of individual studies resulted in pooled sensitivity estimates ranging from 0.79 to 0.84, indicating that no single study exerted undue influence on the overall result.

Similarly, leave‐one‐out analysis for the pooled proportion of CP (Figure S4) demonstrated stable estimates ranging from 1% to 4%, with overlapping confidence intervals across all iterations.

## 4 Discussion

In this systematic review and meta‐analysis, we synthesized evidence from observational studies evaluating the prognostic value of amplitude‐integrated EEG and cUS in neonates with HIE. By integrating incidence estimates of adverse NDO and CP with diagnostic performance measures, this study provides an updated quantitative overview of bedside neurodiagnostic tools commonly used in neonatal intensive care settings.

### 4.1 Interpretation of Main Findings

The pooled incidence of adverse NDO observed across included studies underscores the substantial long‐term burden associated with HIE, even in the era of modern neonatal care. This finding aligns with previous reports indicating that a considerable proportion of neonates with HIE develop unfavorable neurological outcomes despite early intervention. The absence of statistically significant differences in adverse outcome proportions across index test modalities suggests that both neurophysiological and ultrasound‐based assessments capture overlapping aspects of early brain injury. However, this subgroup result should not be interpreted as evidence of direct equivalence between aEEG, cUS, and conventional EEG‐based measures, because most included studies did not perform head‐to‐head comparisons within the same cohort. Rather, the finding indicates that adverse outcomes were commonly observed across studies evaluating different bedside prognostic markers.

Amplitude‐integrated EEG demonstrated strong overall diagnostic performance for predicting adverse NDO, supporting its role as a valuable early prognostic tool. Abnormal background patterns (particularly BS, CLV, and flat tracing), absent or immature SWC, and seizure activity detected by aEEG likely reflect the severity and extent of functional cerebral impairment following hypoxic–ischemic injury (Meder et al. 2022; Gonzalez‐Tamez et al. 2025; Toet et al. 2005). Among these features, background pattern was the most consistently reported and was therefore prioritized for quantitative diagnostic synthesis. SWC and seizure activity are clinically important prognostic features, but their inconsistent reporting across the included studies limited feature‐specific pooled analyses. These results are consistent with prior studies highlighting the association between early aEEG abnormalities and long‐term neurological sequelae (Kota et al. 2024; Das et al. 2021).

For cUS prognostic information depends on both the anatomical region involved and the severity of the detected injury. Abnormalities involving deep gray matter structures, particularly the thalami and basal ganglia, extensive bilateral parenchymal echogenicity, marked loss of gray–white matter differentiation, cystic evolution, cerebral atrophy, or ventriculomegaly were considered more severe structural findings and were harmonized as adverse or severe imaging abnormalities. In contrast, focal or transient echogenicity without deep gray matter involvement or destructive change was considered non‐severe or mild. This distinction is clinically important because mild or transient cUS abnormalities may be less specific, whereas persistent or destructive abnormalities are more likely to reflect established hypoxic–ischemic injury.

The pooled incidence of CP among neonates with HIE was comparatively low, reflecting improvements in perinatal management and neuroprotective strategies (van Oldenmark et al. 2025; Perlman 2006). Nevertheless, CP remains a clinically meaningful outcome due to its lifelong impact on affected individuals and families (Thoresen et al. 2010). The stability of CP estimates across sensitivity analyses further supports the robustness of this finding. The pooled incidence of CP among neonates with HIE was comparatively low, reflecting improvements in perinatal management and neuroprotective strategies (van Oldenmark et al. 2025; Perlman 2006). Nevertheless, CP remains a clinically meaningful outcome due to its lifelong impact on affected individuals and families (Thoresen et al. 2010). The stability of CP estimates across sensitivity analyses further supports the robustness of this finding. However, CP represents only one component of adverse neurodevelopmental outcome. Cognitive impairment, motor dysfunction without a formal CP diagnosis, global developmental delay, severe neurosensory impairment, and death were also included in the broader composite outcome when reported by the original studies.

### 4.2 Heterogeneity and Methodological Considerations

Substantial heterogeneity was observed in analyses of adverse NDO, which is not unexpected given differences in study design, outcome definitions, follow‐up duration, and assessment timing (Pal et al. 2016; Kali et al. 2016; Bedetti et al. 2025). Although meta‐regression analyses did not identify publication year or assessment time point as significant contributors to heterogeneity, residual variability likely reflects unmeasured factors such as therapeutic hypothermia protocols, scoring systems for aEEG interpretation, and variations in neurodevelopmental assessment tools (Salamah et al. 2023; Glass et al. 2023). In addition, heterogeneity likely arose from differences in the specific aEEG features reported, the cUS brain regions assessed, the severity thresholds used to define abnormal ultrasound findings, and the criteria used to classify adverse NDO.

Moderate heterogeneity in diagnostic performance analyses of aEEG suggests that the timing of recording, classification criteria, and clinical context may influence prognostic accuracy. Early recordings may reflect transient physiological disturbances, whereas later assessments may better capture established injury patterns (Abbasi et al. 2020; Matthews et al. 2025). These considerations highlight the importance of standardized protocols and cautious interpretation of aEEG findings in clinical practice. Similarly, cUS findings evolve over time, and the timing of ultrasound relative to the hypoxic–ischemic insult may influence the visibility of parenchymal echogenicity, gray–white matter differentiation, cystic change, atrophy, and ventriculomegaly. Differences in operator experience and reporting standards may further contribute to variability in cUS prognostic performance.

The definition of adverse neurodevelopmental outcome also varied substantially across studies. Some studies used standardized developmental instruments such as the Bayley Scales, whereas others relied on clinical neurological examination, CP diagnosis, global developmental delay, severe disability, or death. To enable synthesis, we harmonized these outcomes into favorable versus adverse categories using study‐specific thresholds and raw outcome counts. Therefore, the pooled adverse outcome proportion should be interpreted as an estimate of the overall burden of unfavorable neurodevelopmental status after HIE, not as an estimate of a single identical developmental endpoint. This approach improves cross‐study comparability but cannot fully eliminate residual misclassification or heterogeneity.

### 4.3 Clinical Implications

From a clinical perspective, the findings support the continued use of aEEG as a valuable component of multimodal neuroprognostication in neonates with HIE. While cUS remains an accessible and practical tool, particularly in settings where advanced imaging is unavailable, its prognostic value appears complementary rather than definitive (Kota et al. 2024; Han et al. 2019; Barth et al. 2020). This may be attributable, in part, to the limited sensitivity of cUS for detecting subtle cortical gray matter injury and early white matter changes compared with magnetic resonance imaging, whereas deep gray matter abnormalities (e.g., thalamic and basal ganglia echogenicity) are more readily identified (Backman et al. 2017; Mathieson et al. 2024). Moderate heterogeneity in diagnostic performance analyses of aEEG suggests that timing of recording, classification criteria, and clinical context may influence prognostic accuracy. Variability in cUS prognostic accuracy may also reflect differences in the specific brain regions evaluated (cortical versus deep gray matter versus white matter), the timing of imaging relative to injury evolution, and operator‐dependent interpretation of injury patterns such as parenchymal echogenicity and gray–white matter differentiation (Salamah et al. 2023; Glass et al. 2023).

In practice, a tiered approach may optimize prognostication: early aEEG (within 6–72 h) provides continuous functional monitoring and risk stratification; cUS (at 24–96 h) offers rapid structural screening, particularly for deep gray matter injury where it shows reasonable concordance with MRI; and MRI (at 5–14 days) serves as the anatomical reference standard for definitive injury characterization in high‐risk or equivocal cases. When MRI is accessible, aEEG and cUS should be viewed as bedside triage tools that inform the timing and interpretation of advanced imaging, rather than standalone substitutes. In resource‐limited settings where MRI is unavailable, concordant severe abnormalities on both bedside modalities may provide sufficient prognostic information to guide clinical decisions (Chau et al. 2014). Nevertheless, prognostic decisions should not rely on a single test result in isolation; rather, they should integrate clinical encephalopathy severity, serial neurological examination, aEEG evolution, cUS findings, MRI when available, and standardized follow‐up assessment.

### 4.4 Strengths and Limitations

This study has several strengths, including a comprehensive search strategy across multiple databases, strict inclusion criteria focusing on term and near‐term neonates with HIE, and the use of robust statistical methods to account for between‐study heterogeneity. Sensitivity analyses further supported the stability of pooled estimates. A further strength of the analysis is the explicit definition and harmonization of aEEG features, cUS injury patterns, cUS severity categories, and adverse neurodevelopmental outcome measures, which improves transparency and interpretability.

However, several limitations should be acknowledged. First, the included studies were observational in nature, which may introduce residual confounding. Second, variability in outcome definitions and follow‐up durations may limit direct comparability across studies. Third, the number of studies contributing to certain analyses, particularly CP outcomes, was limited, reducing statistical power for subgroup and meta‐regression analyses. Fourth, the pooled adverse outcome estimate was based on harmonized study‐level definitions rather than individual patient data or identical developmental instruments, which may have introduced residual misclassification. Fifth, cUS findings were less frequently reported than aEEG findings, and the available cUS data differed in anatomical regions assessed, injury patterns described, and severity thresholds used. Sixth, SWC and seizure activity were not consistently reported across aEEG studies, limiting feature‐specific quantitative analyses beyond background pattern. Finally, subgroup comparisons between aEEG, cUS, and conventional EEG‐based measures were indirect and should not be interpreted as direct comparative test accuracy.

### 4.5 Future Directions

Future research should prioritize standardized reporting of aEEG patterns, uniform neurodevelopmental outcome measures—ideally employing validated tools such as the Bayley Scales at predefined time points (e.g., 18–24 months corrected age) with consistent severity thresholds—and longer‐term follow‐up to improve comparability across studies. Prospective multicenter cohorts integrating neurophysiological, neuroimaging, and biomarker data may further enhance early prognostic accuracy. In addition, evaluating prognostic tools within the context of contemporary therapeutic hypothermia protocols remains an important area for ongoing investigation. Such studies should ideally combine serial aEEG, structured cUS, MRI, and standardized long‐term neurodevelopmental assessment to develop validated multimodal prediction models.

## 5 Conclusion

This systematic review and meta‐analysis demonstrate that adverse NDO remain common among term and near‐term neonates with HIE. aEEG shows strong prognostic value for early prediction of unfavorable NDO, supporting its role as an important bedside neurophysiological tool in neonatal intensive care settings. This prognostic value was most consistently supported for abnormal aEEG background patterns, particularly BS, CLV, and flat/isoelectric tracing, while SWC and seizure activity require more standardized reporting in future studies. cUS, while widely accessible and clinically practical, appears to provide complementary rather than definitive prognostic information.

The findings highlight the importance of integrating neurophysiological monitoring with clinical assessment and structural imaging to achieve a more comprehensive approach to early neuroprognostication in HIE. Given the observed heterogeneity across studies, standardized assessment protocols and uniform outcome definitions are needed to enhance comparability and clinical interpretability. In particular, future studies should apply explicit definitions of aEEG features, structured cUS severity grading, and prespecified neurodevelopmental outcome thresholds.

Future well‐designed prospective studies with standardized methodologies and long‐term follow‐up are warranted to refine prognostic models and optimize early risk stratification, ultimately improving individualized care and long‐term outcomes for neonates affected by HIE. Prospective studies integrating serial aEEG, structured cUS, MRI, and standardized neurodevelopmental follow‐up are particularly needed to validate multimodal prediction models and improve the clinical utility of bedside prognostic tools.

## Funding

The authors have nothing to report.

## Ethics Statement

The authors have nothing to report.

## Conflicts of Interest

The authors declare no conflicts of interest.

## Supporting information


**Figure S1:** Subgroup analysis of adverse neurodevelopmental outcomes stratified by index test modality.

**Figure S2:** Leave‐one‐out sensitivity analysis for pooled adverse neurodevelopmental outcomes.

**Figure S3:** Leave‐one‐out sensitivity analysis for diagnostic sensitivity of amplitude‐integrated EEG.

**Figure S4:** Leave‐one‐out sensitivity analysis for pooled incidence of cerebral palsy.

**Data S1:** Supporting Information.

**Table S1:** Index‐test features and harmonized abnormality categories across included studies.
**Table S2:** Outcome definitions and harmonized adverse outcome categories across included studies.

## Data Availability

The data that support the findings of this study are available from the corresponding author upon reasonable request.
