# Supplementary material for: Comparison of Cranial Ultrasound and Amplitude‐Integrated Electroencephalography in Predicting Neurodevelopmental Outcomes in Neonates With Hypoxic–Ischemic Encephalopathy: A Systematic Review and Meta‐Analysis
Source: J Clin Ultrasound. 2026 Jul 17;54(9):2193–207. doi: 10.1002/jcu.70340 (PMC13730180; doi:10.1002/jcu.70340)
Supplement: Supplementary file 1 [file JCU-54-2193-s001.docx]

# Literature Search Strategy Report

## Objective

To identify evidence on the prognostic value of amplitude-integrated EEG (aEEG), conventional EEG, and cranial/cerebral ultrasound for neurodevelopmental and clinical outcomes in neonatal hypoxic-ischemic encephalopathy (HIE).

## Design Principles

Sensitivity prioritized over excessive restriction to minimize missed evidence.

Controlled vocabulary (where available) is combined with free-text synonyms.

American and British spellings are both included (“ischemic” and “ischaemic”).

The wildcard predict* is retained to capture variants such as “predictive” and “prediction”.

No restriction on timing of outcome assessment is applied to avoid over-narrowing the evidence base.

## PubMed

### Search strategy

("hypoxic ischemic encephalopathy"
 OR "hypoxic ischaemic encephalopathy"
 OR HIE
 OR "birth asphyxia")
AND
("amplitude integrated EEG"
 OR aEEG
 OR EEG
 OR "cranial ultrasound"
 OR "cerebral ultrasound")
AND
("neurodevelopmental outcome"
 OR prognosis
 OR predict*
 OR mortality
 OR "cerebral palsy")

### Rationale

American and British spellings are explicitly included.

The wildcard term supports broader capture of prediction-related language.

Outcome timing is intentionally unrestricted to preserve sensitivity.

## Embase

### Search strategy

('hypoxic ischemic encephalopathy'/exp
 OR 'hypoxic ischaemic encephalopathy'
 OR HIE
 OR 'birth asphyxia'/exp)
AND
('amplitude integrated electroencephalography'
 OR aEEG
 OR EEG/exp
 OR 'cranial ultrasound'/exp
 OR 'cerebral ultrasound')
AND
('neurodevelopmental outcome'/exp
 OR prognosis/exp
 OR predict*
 OR mortality/exp
 OR 'cerebral palsy'/exp)

### Rationale

/exp is used to explode EMTREE hierarchies.

High-frequency Embase subject headings (e.g., EEG, mortality, cerebral palsy) are explicitly leveraged.

Free-text terms are preserved to capture recently indexed or incompletely indexed records.

## Web of Science Core Collection

### Search strategy (Topic field)

TS=(
 ("hypoxic ischemic encephalopathy"
 OR "hypoxic ischaemic encephalopathy"
 OR HIE
 OR "birth asphyxia")
 AND
 ("amplitude integrated EEG"
 OR aEEG
 OR EEG
 OR "cranial ultrasound"
 OR "cerebral ultrasound")
 AND
 ("neurodevelopmental outcome"
 OR prognosis
 OR predict*
 OR mortality
 OR "cerebral palsy")
)

### Rationale

Web of Science does not rely on MeSH or EMTREE; the Topic field (TS) is used consistently.

TS searches titles, abstracts, author keywords, and KeyWords Plus.

Logical structure is aligned with PubMed to support PRISMA reproducibility.

## Cochrane Library (CENTRAL)

### Search strategy

("hypoxic ischemic encephalopathy"
 OR "hypoxic ischaemic encephalopathy"
 OR HIE
 OR "birth asphyxia")
AND
("amplitude integrated EEG"
 OR aEEG
 OR EEG
 OR "cranial ultrasound"
 OR "cerebral ultrasound")
AND
("neurodevelopmental outcome"
 OR prognosis
 OR predict*
 OR mortality
 OR "cerebral palsy")

### Rationale

Although CENTRAL is RCT-focused, retaining this strategy supports comprehensive identification of early cohorts and nested studies.

No additional study-design filters are applied to avoid excluding relevant non-randomized original research.

## Notes for Reporting (PRISMA Alignment)

The same three-concept structure (population/condition, index tests, outcomes) is maintained across databases.

Database-specific indexing features are used when available, while preserving conceptual equivalence.

This approach balances reproducibility, transparency, and retrieval sensitivity.
