# Supplementary material for: Comparison of Cranial Ultrasound and Amplitude‐Integrated Electroencephalography in Predicting Neurodevelopmental Outcomes in Neonates With Hypoxic–Ischemic Encephalopathy: A Systematic Review and Meta‐Analysis
Source: J Clin Ultrasound. 2026 Jul 17;54(9):2193–207. doi: 10.1002/jcu.70340 (PMC13730180; doi:10.1002/jcu.70340)
Supplement: Supplementary file 2 [file JCU-54-2193-s003.docx]

**Supplementary Table S1. Index-test features and harmonized abnormality categories across included studies**

| **Study (Year)** | **Index test** | **Main features assessed** | **Original abnormality/severity definition** | **Harmonized category** |
| --- | --- | --- | --- | --- |
| Hellstrom-Westas (1995)[21] | aEEG | Background pattern | Abnormal voltage/continuity; BS, CLV, or FT considered severe | Abnormal aEEG background |
| Toet (1999)[22] | aEEG | Background pattern at 3 and 6 h | Normal, moderately abnormal, or severely abnormal background | Severe or non-recovering background = abnormal |
| Shany (2006)[23] | aEEG | Background pattern and voltage | Study-defined abnormal pattern/voltage | Study-defined abnormal aEEG |
| Thoresen (2010)[24] | aEEG | Early background pattern | Severe or persistent background abnormality | Severe abnormal background |
| Annink (2020)[13] | cUS | Cortical gray matter, deep gray matter, white matter, ventricles | Structured cUS score assessing injury topography and severity | Moderate/severe cUS injury |
| Sewell (2018)[25] | aEEG | Background evolution ≤72 h | Abnormal or non-recovering background evolution | Persistent abnormal aEEG |
| Meder (2022)[9] | aEEG | Longitudinal background pattern | Study-defined abnormal background trajectory | Abnormal aEEG trajectory |
| Cornet (2025)[6] | EEG/BSN | EEG background and BSN features | Automated EEG-defined abnormal background/BSN | Abnormal EEG/BSN |
| Gonzalez-Tamez (2025)[26] | EEG/BSN | Early EEG background trend | Automated EEG-defined abnormal trend | Abnormal EEG trend |
| Kota (2024)[27] | EEG/BSN | Quantitative EEG features | Study-defined quantitative EEG abnormality | Abnormal qEEG background |

**Notes:**

aEEG, amplitude-integrated electroencephalography; BS, burst suppression; BSN, burst suppression network; CLV, continuous low voltage; cUS, cranial ultrasound; EEG, electroencephalography; FT, flat tracing; qEEG, quantitative electroencephalography.

**Supplementary Table S2. Outcome definitions and harmonized adverse outcome categories across included studies**

| **Study (Year)** | **Outcome/reference standard** | **Follow-up** | **Original adverse outcome definition** | **Harmonized category** |
| --- | --- | --- | --- | --- |
| Hellstrom-Westas (1995)[21] | Clinical neurological outcome | 12–72 mo | Death or neurological sequelae, including CP | Death or neurological sequelae |
| Toet (1999)[22] | Clinical/developmental outcome | 12 mo–6 y | Death, CP, or global delay; DQ <80 | Death, CP, or developmental delay |
| Shany (2006)[23] | Combined neurodevelopmental outcome | ≥30 mo | Death or study-defined poor outcome | Death or poor outcome |
| Thoresen (2010)[24] | Bayley-II and GMFCS | 18 mo | Death or severe disability | Death or severe disability |
| Annink (2020)[13] | BSID-III and clinical diagnosis | 24 mo | Death, CP, or BSID-III <85 | Death, CP, or BSID-III <85 |
| Sewell (2018)[25] | MRI injury/death | Neonatal period | Death or severe MRI injury | Death or severe MRI injury* |
| Meder (2022)[9] | Bayley-II | 24 mo | Death or MDI/PDI <70 | Death or Bayley-II <70 |
| Cornet (2025)[6] | Bayley-III and GMFCS | 22–36 mo | Death or severe NDI | Death or severe NDI |
| Gonzalez-Tamez (2025)[26] | Clinical/developmental outcome | 24 mo | Moderate–severe NDI or death | Death or moderate–severe NDI |
| Kota (2024)[27] | Bayley-III | 24 mo | Death or Bayley-III <85 | Death or Bayley-III <85 |

**Notes:**

BSID-III, Bayley Scales of Infant and Toddler Development, Third Edition; CP, cerebral palsy; DQ, developmental quotient; GMFCS, Gross Motor Function Classification System; MDI, Mental Development Index; MRI, magnetic resonance imaging; NDI, neurodevelopmental impairment; PDI, Psychomotor Development Index. mo: months; y: years.
